# Heated High-Flow Nasal Cannula Therapy for Pediatric Obstructive Sleep Apnea: Physiology, Clinical Evidence, and Future Directions

**DOI:** 10.3390/children13081027

**Published:** 2026-08-01

**Authors:** Natalia S. Escobar, Reshma Amin

**Affiliations:** 1 Division of Respiratory Medicine, Department of Pediatrics, The Hospital for Sick Children, University of Toronto, Toronto, ON M5G 1X8, Canada; reshma.amin@sickkids.ca; 2 Child Health and Evaluative Science, SickKids Research Institute, Toronto, ON M5G 0A4, Canada; 3 Department of Paediatrics, University of Toronto, Toronto, ON M5G 1X8, Canada

**Keywords:** pediatric obstructive sleep apnea, heated high-flow nasal cannula, continuous positive airway pressure, pediatric sleep medicine

## Abstract

**Highlights:**

**What are the main findings?**

• Heated high-flow nasal cannula therapy can reduce obstructive respiratory events and improve oxygenation in selected children with obstructive sleep apnea, particularly when conventional positive airway pressure is not tolerated or feasible.
• Its physiological effects are multifactorial and include flow-dependent positive airway pressure, nasopharyngeal dead-space washout, reduced inspiratory resistance, and improved ventilatory efficiency; however, treatment response varies considerably with flow, leak, interface fit, airway anatomy, and patient size.

**What are the implications of the main findings?**

• Heated high-flow nasal cannula should be considered a second-line or complementary therapy for carefully selected children rather than a universal substitute for CPAP or BPAP.
• Standardized titration protocols, objective adherence monitoring, and prospective multicentre studies are needed to define optimal patient selection, long-term effectiveness, safety, and clinically meaningful outcomes.

**Abstract:**

Pediatric obstructive sleep apnea (OSA) is a common disorder associated with significant neurocognitive, behavioral, cardiovascular, and metabolic consequences. Although adenotonsillectomy remains first-line therapy for many children, residual OSA is common, particularly among those with obesity, craniofacial abnormalities, genetic syndromes, neuromuscular disease, or other forms of medical complexity. Continuous positive airway pressure (CPAP) is the standard non-surgical treatment; however, long-term effectiveness is frequently limited by poor tolerance and adherence. Heated high-flow nasal cannula (HFNC) therapy has emerged as a potential alternative for selected children with sleep-disordered breathing, particularly those who are unable to tolerate conventional positive airway pressure therapy. Unlike CPAP, HFNC delivers heated, humidified gas through an open nasal interface and may improve sleep-disordered breathing through a combination of flow-dependent positive airway pressure generation, dead-space washout, improved ventilatory efficiency, enhanced gas conditioning, and reductions in inspiratory resistance. However, the relative contribution of these mechanisms during sleep remains incompletely understood. Current clinical evidence consists primarily of physiological studies, retrospective cohorts, case series, and a limited number of prospective comparative studies. Collectively, these data suggest that HFNC can reduce obstructive respiratory events and improve oxygenation in selected pediatric populations, including children with persistent OSA, CPAP intolerance, medical complexity, and syndromic conditions. Nevertheless, important uncertainties remain regarding optimal patient selection, titration strategies, patient monitoring, long-term adherence and comparative effectiveness relative to CPAP. This review summarizes the physiological basis of HFNC therapy, critically appraises the current clinical evidence, discusses practical considerations related to adherence and implementation, and highlights key knowledge gaps and future research priorities. Overall, HFNC should be viewed as an alternative for selected children who cannot tolerate CPAP, rather than as a universal substitute for pressure-based therapy.

## 1. Introduction

Pediatric obstructive sleep apnea (OSA) is characterized by recurrent partial or complete upper airway obstruction during sleep, resulting in intermittent hypoxemia, sleep fragmentation, and increased work of breathing [1]. The estimated prevalence of OSA in otherwise healthy children is approximately 1–5%, but it is substantially higher among children with obesity, craniofacial abnormalities, genetic syndromes, neuromuscular disease, and other forms of medical complexity [2,3,4,5]. Untreated OSA is associated with important adverse consequences, including neurocognitive and behavioral difficulties, impaired quality of life, cardiovascular morbidity, metabolic dysfunction, and increased healthcare utilization [6,7,8,9].

The management of pediatric OSA includes both medical and surgical approaches and should be individualized according to the underlying pathophysiology, severity of disease, and patient characteristics [1]. In many children, OSA is driven by adenotonsillar hypertrophy; therefore, adenotonsillectomy is generally considered the first-line treatment when adenotonsillar hypertrophy is present [1,10,11]. Although adenotonsillectomy improves OSA severity in many children, residual or persistent OSA remains common, particularly among children with obesity, craniofacial disorders, Down syndrome, neuromuscular disease, or broader medical complexity [11,12,13,14]. For these children, and for those in whom surgery is not indicated or is insufficient, positive airway pressure therapy, most commonly continuous positive airway pressure (CPAP), remains the main nonsurgical treatment option [1,15].

CPAP is highly efficacious when used, but its effectiveness in children is frequently limited by intolerance and poor adherence [16,17,18,19]. Barriers to its use include discomfort with the mask interface, interface leak due to improper fit or change in sleeping position, skin irritation or breakdown, nasal irritation, edema and bleeding, behavioral intolerance, and the need for time-intensive acclimatization [18,20]. In infants and young children, concerns have also been raised about potential midface and dental effects from the use of chronic positive-pressure mask interfaces during a period of facial bone development [21,22]. Consequently, many children with persistent OSA remain undertreated, highlighting the need for alternative, effective, and well-tolerated non-invasive respiratory support strategies.

Heated high-flow nasal cannula (HFNC) was initially developed and adopted primarily as a form of non-invasive respiratory support for neonates and children with acute respiratory illness [23]. Its use subsequently expanded across neonatal, pediatric, and adult care because it can deliver heated and humidified gas at flows that meet or exceed the patient’s inspiratory demand through a relatively comfortable open nasal interface. In current pediatric practice, HFNC is most commonly used for acute hypoxemic respiratory failure, bronchiolitis, post-extubation respiratory support, and selected neonatal respiratory disorders [24]. Its application in chronic respiratory support and sleep-disordered breathing is more recent and remains less standardized.

HFNC therapy has emerged as a potential alternative for selected children with sleep-disordered breathing, particularly those who are intolerant of CPAP or in whom mask-based therapy is not feasible [14,25,26,27]. HFNC delivers heated, humidified air or air-oxygen mixtures through a soft, loose-fitting nasal cannula using an open system, instead of a tight mask seal as in CPAP. Proposed mechanisms of action include washout of nasopharyngeal dead space, reduction in inspiratory resistance, generation of flow-dependent positive airway pressure, improved gas conditioning, reduced work of breathing, and potential stabilization of the upper airway during sleep [28,29,30,31].

The role of HFNC in pediatric sleep medicine is evolving rapidly. Early studies suggest that HFNC may improve obstructive respiratory events and oxygenation in selected children with OSA, including those unable to tolerate conventional positive airway pressure therapy; however, important questions remain regarding its mechanisms of action, efficacy across different patient populations, long-term outcomes, safety, adherence, and optimal implementation [25,26,27,32]. In this review, we describe the current evidence supporting the use of HFNC in pediatric sleep-disordered breathing and discuss future directions for research and clinical care.

## 2. Literature Search Strategy

A narrative literature search was conducted to identify studies evaluating the physiological mechanisms, clinical efficacy, adherence, safety, and home implementation of HFNC in pediatric obstructive sleep apnea and sleep-disordered breathing. MEDLINE/PubMed and Embase were searched from database inception to 10 June 2026. Search terms included combinations of “high-flow nasal cannula,” “heated high flow,” “nasal high flow,” “pediatric,” “child,” “infant,” “obstructive sleep apnea,” “sleep-disordered breathing,” “continuous positive airway pressure,” “physiology,” “adherence,” and “home ventilation.” Reference lists of relevant articles and reviews were also screened to identify additional publications. Original pediatric studies, physiological and experimental studies relevant to the proposed mechanisms of HFNC, systematic reviews, and selected adult studies providing mechanistic context were included. Articles were excluded if they did not address HFNC physiology or its application to sleep-disordered breathing, were not available in English, or consisted solely of conference abstracts without sufficient methodological or outcome data. Given the narrative nature of this review, study selection and synthesis were not conducted according to a formal systematic-review protocol.

### 2.1. Summary of the Evidence

#### 2.1.1. Physiological Mechanisms of HFNC During Sleep

**Figure 1:**
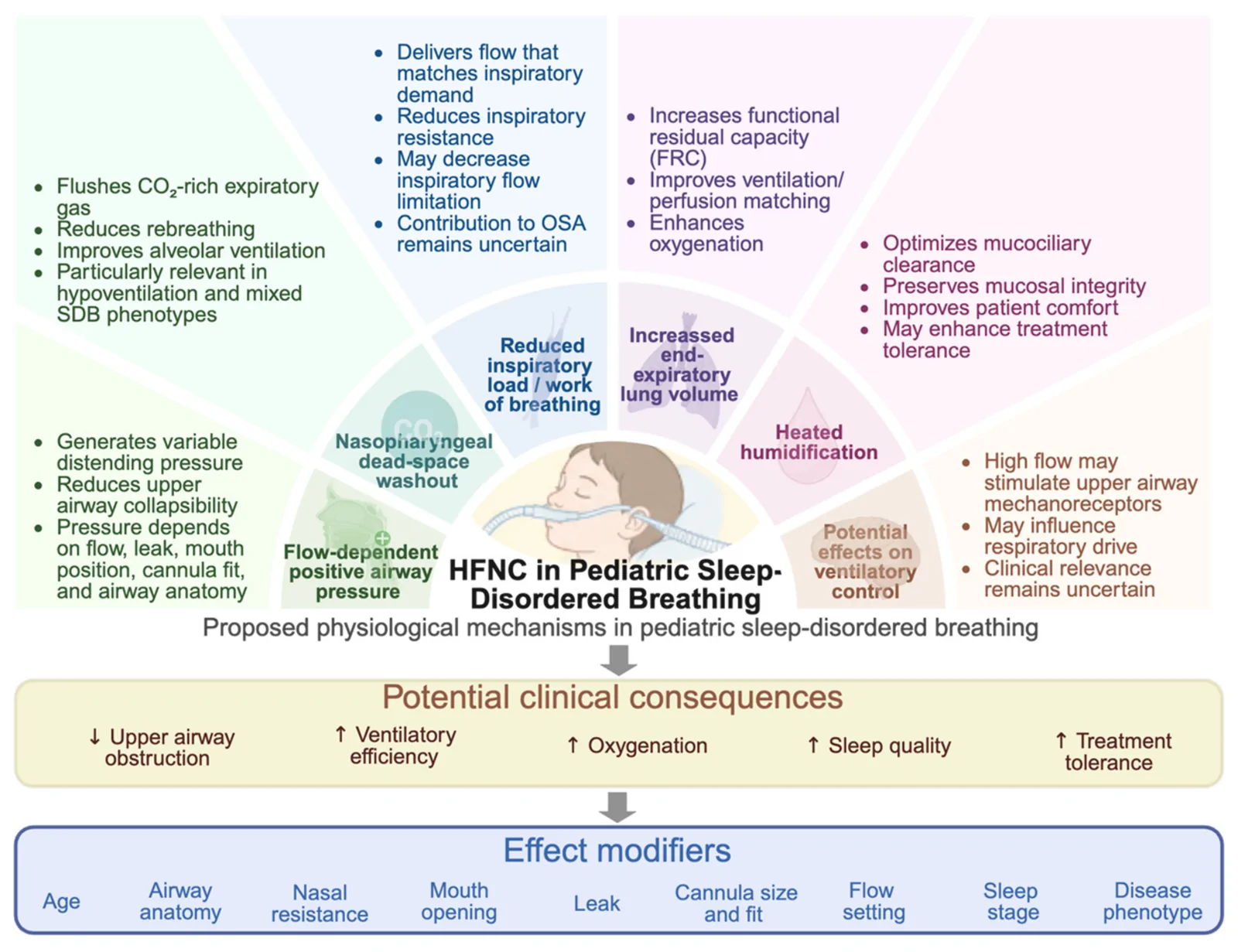
Proposed mechanisms of action of heated high-flow nasal cannula therapy in pediatric sleep-disordered breathing.

The proposed physiological rationale for the use of HFNC therapy in pediatric sleep-disordered breathing is multifactorial and differs substantially from conventional positive airway pressure therapies (see Figure 1). Unlike CPAP, which delivers a prescribed and measurable distending pressure, HFNC is an open system in which physiological effects are flow- and interface-dependent and highly influenced by leak. Proposed mechanisms include generation of variable positive airway pressure, dead-space washout, reduction in inspiratory resistance, improved gas conditioning, and enhanced ventilatory efficiency [23,30].

Several anatomical and physiological characteristics may make children particularly responsive to HFNC, although direct pediatric–adult comparative studies are lacking. Children have smaller upper-airway dimensions and distinct developmental airway mechanics, relatively high airway resistance, a proportionally greater contribution of tonsillar and adenoidal tissue to upper-airway narrowing, and a greater propensity for dynamic pharyngeal narrowing during sleep [33,34]. Consequently, even modest reductions in inspiratory resistance or small increases in distending pressure may have proportionally greater effects on upper airway patency than in adults. Children also have smaller upper-airway dead-space volumes and higher respiratory rates relative to body size; therefore, the relative contribution of dead-space washout may differ according to age, airway dimensions, breathing pattern, and flow relative to body size [35,36,37]. Conversely, the pressure generated remains highly dependent on mouth position, cannula fit, leak, airway anatomy, and flow relative to body size. Thus, these characteristics provide a plausible physiological rationale for pediatric use but do not establish that HFNC is more effective in children than in adults.

However, the mechanisms by which HFNC improves obstructive sleep-disordered breathing in children remain unclear. Much of the physiological rationale has been extrapolated from acute respiratory care studies, neonatal data, experimental airway models, and adult physiological investigations, rather than from mechanistic studies performed during sleep in children with OSA.

The most relevant proposed mechanism is the generation of flow-dependent positive airway pressure. This pressure is thought to arise, at least in part, from expiratory resistance while breathing against high inspiratory flows, thereby providing a low-level pneumatic splint that may reduce upper airway collapsibility during sleep [31,35,38,39]. Pediatric model studies support the concept that HFNC can generate clinically meaningful distending pressure but also demonstrate that this effect is highly variable.

Gray et al. used anatomically accurate 3D-printed pediatric airway models, representing a preterm neonate, term neonate, toddler, and small child, connected to a computerized spontaneous breathing lung model [38]. In closed-mouth conditions, approximately 6 cmH_2_O of tracheal pressure was achieved at 6–8 L/min in neonatal models and at approximately 12–20 L/min in older pediatric models. At the highest tested flows, tracheal pressures reached 7–10 cmH_2_O in neonatal and toddler models and up to 24 cmH_2_O in the small-child model; mouth opening reduced generated pressure by at least 50% [38].

Similarly, Ejiofor et al. evaluated PEEP generation across pediatric lung models from 1 month to 18 years using HFNC flows of 6–60 L/min and simulated leak conditions of 25%, 50%, and 75% [35]. HFNC generated end-expiratory alveolar pressures ranging from 1.2 to 36 cmH_2_O, with higher pressures observed as flow increased and leak decreased. These data reinforce that HFNC can generate substantial positive pressure under specific conditions, but that delivered pressure is strongly influenced by leak, cannula size, patient weight, and model characteristics. Importantly, the pressure generated by HFNC during sleep in children with OSA has not been directly measured, largely because of the technical challenges of assessing pharyngeal pressure during polysomnography. Moreover, pressures generated under clinically relevant open-system conditions may be considerably lower than those achieved in closed-mouth models.

These pressure effects may have consequences beyond upper airway splinting. Even modest increases in end-expiratory pressure may increase functional residual capacity, improve ventilation–perfusion matching, and enhance oxygenation, mechanisms that are well described in acute respiratory support literature [29,30,40]. In children with sleep-disordered breathing, these effects may contribute to improvements in oxygen saturation, particularly in those with obesity, atelectasis-prone physiology, chronic lung disease, or hypoventilation phenotypes. However, whether recruitment of end-expiratory lung volume meaningfully contributes to improvements in obstructive events during sleep remains uncertain.

Nasopharyngeal dead-space washout is another consistently described physiological effect of HFNC [30,41,42,43]. In upper airway models, Möller et al. showed that nasal high flow rapidly cleared tracer gas from the nasal cavities in a flow-dependent manner, with clearance half-times generally below 1 s and complete nasal cavity clearance within approximately 1 s in an anatomically based model. Clearance increased by approximately 1.8 mL/s for each 1 L/min increase in flow [42]. By reducing rebreathing of carbon dioxide-rich expired gas, this mechanism may improve ventilatory efficiency, particularly in hypoventilation-prone or mixed sleep-disordered breathing phenotypes; its contribution to improvement in purely obstructive OSA remains less certain.

More recently, asymmetrical nasal high-flow interfaces have been developed to enhance the physiological effects of HFNC [44]. These cannulas use unequal prong sizes to increase differential nare occlusion, reduce leak, and promote reverse flow through the nasopharynx, thereby augmenting expired-gas clearance and airway pressure generation compared with conventional symmetrical interfaces [45,46]. However, pediatric data are currently lacking, and the relevance of asymmetrical interfaces for children with OSA or other forms of sleep-disordered breathing remains unknown.

Human physiological data are consistent with these findings, while also clarifying what HFNC may not do. In awake adults studied in the supine position, HFNC flows up to 60 L/min generated approximately 7 cmH_2_O of positive airway pressure, reduced breathing frequency, increased expiratory time, and did not increase genioglossus muscle activity [31]. A neuromuscular contribution remains biologically plausible, as high inspiratory flow and associated pressure fluctuations could theoretically activate upper airway mechanoreceptors and reflexively augment pharyngeal dilator muscle tone, including genioglossus activity [47]. However, in the same physiological study, neither tonic nor peak genioglossus activity increased significantly with higher HFNC flows [31]. These findings suggest that HFNC is unlikely to improve upper airway stability primarily through recruitment of pharyngeal dilator muscles. Because the study was performed during wakefulness, in adults, and largely in individuals without OSA, the possibility of reflex neuromuscular effects during sleep in children with obstructive sleep-disordered breathing remains unresolved.

Whether reductions in inspiratory resistance and work of breathing contribute meaningfully to improvements in pediatric OSA remains uncertain. Although HFNC may reduce inspiratory loading by delivering flow directly at the nares and decreasing entrainment through a narrowed upper airway, most supporting evidence comes from acute respiratory failure, neonatal respiratory support, and adult physiological studies rather than from mechanistic studies conducted during sleep in children with OSA [48,49].

#### 2.1.2. Clinical Evidence for HFNC in Pediatric Sleep-Disordered Breathing

Clinical evidence supporting HFNC therapy for pediatric sleep-disordered breathing remains limited but has grown over the past decade. The available literature includes early proof-of-concept physiological studies, retrospective cohorts, case series, short-term polysomnographic titration studies, and two comparative crossover studies: one randomized laboratory crossover trial and a more recent prospective study with paired laboratory titrations followed by a randomized home crossover phase. Across these studies, HFNC has generally been evaluated not as first-line therapy for uncomplicated pediatric OSA, but as an alternative for children with persistent OSA, medical complexity, craniofacial or syndromic conditions, young age, poor surgical candidacy, or intolerance of conventional positive airway pressure therapy [25,26,27,50,51,52]. See Table 1 for a summary of clinical studies using HFNC for the treatment of pediatric sleep-disordered breathing.

A systematic review and meta-analysis subsequently synthesized the available pediatric evidence and reported significant reductions in the obstructive apnea-hypopnea index (OAHI), obstructive apnea index (OAI), obstructive hypopnea index (OHI), and improvement in oxygen saturation nadir with HFNC therapy. However, interpretation of these findings is limited by the small number of included studies and substantial clinical heterogeneity. Consequently, the meta-analysis supports the signal of therapeutic efficacy but does not resolve questions regarding long-term effectiveness, optimal patient selection, adherence, safety, or comparative effectiveness relative to CPAP [32].

Early studies established proof of concept. McGinley et al. evaluated treatment with nasal insufflation, a precursor of contemporary HFNC systems, in 12 children with mild-to-severe OSA and demonstrated reductions in inspiratory flow limitation, respiratory rate, inspiratory duty cycle (length of the inspiratory time divided by length of the respiratory cycle), arousals, and AHI during sleep [25]. Although improvements approached those previously observed with CPAP in the same cohort, interpretation is limited by the retrospective nature of the CPAP comparison, the absence of a washout period, and the use of a fixed maximum flow of 20 L/min rather than individualized HFNC titration.

Subsequent case series explored HFNC in children with complex causes of OSA. Joseph et al. described five children aged 22 months to 15 years with OSA and heterogeneous comorbidities, including congenital anomalies, hypotonia, psychomotor delay, Treacher Collins syndrome, and a history of prematurity [27]. HFNC was associated with improvements in AHI and oxygenation, supporting feasibility in complex pediatric OSA, but the small sample size and marked heterogeneity limit generalizability.

Evidence has also expanded to infants and young children, a population in whom CPAP implementation may be particularly challenging. In one retrospective titration study of ten infants with OSA, HFNC reduced median OAHI from 9.1 (IQR 5.1–19.3) to 0.9 (IQR 0–1.6; *p* = 0.005) events/h and improved oxygen saturation nadir from 88% (IQR 83–94%) to 94% (IQR 93–96%; *p* = 0.040) [53]. Notably, infants requiring supplemental oxygen in addition to HFNC were excluded from the final analysis, which may limit generalizability to syndromic or medically complex infants. A larger cohort of 22 infants and young children with moderate-to-severe OSA, many of whom were poor surgical candidates, had persistent OSA after surgery, or were unable to tolerate CPAP, showed a reduction in mean OAHI from 28.9 (95% CI 17.6–40.2) to 2.6 (95% CI 1.1–4.0) events/h during HFNC titration [51]. Nineteen children were prescribed home HFNC. By 12 months, four were lost to follow-up and OSA had resolved in three; among the 12 children with ongoing OSA, seven continued HFNC and five discontinued because of intolerance. Cannula dislodgement was the most common complication, with additional reported issues including skin irritation, dry mucous membranes, restlessness, oxygen desaturation, and increased central apneas [51].

Among children with persistent OSA and CPAP intolerance, Hawkins et al. evaluated ten children aged 1–18 years with moderate-to-severe OSA and heterogeneous comorbidities, including Down syndrome, craniofacial syndromes, and obesity [52]. HFNC was titrated during overnight polysomnography using room air, with supplemental oxygen added only if hypoxemia persisted at maximal flow rate based on cannula size [52]. Median OAHI decreased from 11.1 (IQR 8.7–18.8) to 2.1 (IQR 1.7–2.2) events/h, mean SpO2 increased from 91.3% (89.6% to 93.5%) to 94.9% (92.4% to 96.0%; *p* < 0.002), and oxygen desaturation index decreased from 19.2 (12.7–25.8 events/h) to 6.4 events/h (4.7–10.7 events/h; *p* = 0.013) events/h. Importantly, improvements in obstructive indices persisted in the subgroup treated with room air alone, suggesting that the effect was not solely explained by supplemental oxygen [52]. However, the study remained limited by small sample size (*n* = 10), inclusion of two retrospective participants, lack of CPAP comparison, and potential selection bias.

Amaddeo et al. provided clinically important objective adherence data in children with severe OSA who were nonadherent to CPAP [50]. In this cohort of eight children, including six with Down syndrome, one with Pierre Robin sequence, and one with Pfeiffer syndrome, HFNC was used as rescue therapy after CPAP use of less than 2 h/night. Adherence data were derived from the device display, which reported total time divided by the number of days of use, limiting direct comparison with conventional CPAP adherence metrics. After one month, five children used HFNC for more than 4 h/night, with mean (±SD) compliance 7 h 10 min (±0 h 36 min/night). In these children, OSA was corrected, with mean AHI decreasing to approximately 2 (±2) events/h. However, HFNC was not accepted by the three oldest children with Down syndrome, underscoring that improved tolerability is not universal and may vary by age, neurodevelopmental profile, and caregiver-child acceptance [50].

More recent studies have begun to address comparative effectiveness and home implementation. In a randomized crossover trial of 18 children with obesity and/or medical complexity and moderate-to-severe OSA, Fishman et al. found comparable short-term improvements with HFNC and CPAP during titration studies [26]. Mean (±SD) OAHI decreased from 23.1 (±21.7) to 3.3 (±5.0) events/h with HFNC and to 4.3 (±6.3) events/h with CPAP, with no significant difference in the magnitude of reduction between therapies. Improvements in oxygenation, oxygen desaturation index, arousal index, sleep efficiency, and transcutaneous CO_2_ were also comparable. A subsequent analysis of event-related hypoxic burden and pulse rate response suggested that the two therapies may not be physiologically equivalent: both reduced cumulative nocturnal hypoxemia, but only CPAP significantly attenuated pulse rate response to obstructive events, suggesting a potentially greater effect on autonomic stress [54].

A more recent prospective study in otherwise healthy children and adolescents with moderate-to-severe OSA also found similar residual OAHI during HFNC and CPAP titration among 29 participants (mean 5.4 SD ± 12.6 vs. 3.6 ± 7.8 events/h; *p* = 0.281), although CPAP produced greater improvement in oxygen saturation nadir [55]. In the subsequent home crossover phase, adherence was significantly better with CPAP than with HFNC, and HFNC did not provide additional benefit for daytime sleepiness or behavioural outcomes, despite improvement in disease-specific quality of life with both therapies [55]. These findings challenge the assumption that a less intrusive nasal cannula interface necessarily translates into better adherence in older children and adolescents.

Real-world home HFNC data provide a complementary but lower-certainty perspective. In a multicenter retrospective cohort of 75 children prescribed home HFNC, OSA was the most common indication (33 children, 44%) [56]. Caregiver satisfaction based on a self-reported follow-up questionnaire was high. The investigators also compared hospitalization days during the 12 months before and after HFNC initiation and found a reduction following treatment, together with improved weight gain and no serious adverse events. However, polysomnographic efficacy was not evaluated, and these findings should be interpreted as evidence of feasibility and apparent safety rather than disease-specific efficacy for pediatric OSA [56].

Although short-term titration studies suggest that HFNC may produce reductions in OSA severity comparable to CPAP in selected children, the two therapies should not be considered physiologically interchangeable. CPAP provides fixed, titratable pressure and objective monitoring, whereas HFNC delivers variable flow-dependent pressure through an open interface with limited adherence and efficacy downloads. Therefore, HFNC is best viewed as an alternative for selected children who cannot tolerate CPAP, rather than a universal substitute for pressure-based therapy.

**Table 1:** Clinical studies evaluating HFNC for pediatric obstructive sleep apnea.

Study	Design	Population	N	HFNC Intervention	Main Findings	Key Limitations
McGinley et al. [25]	Prospective physiological study	Children with mild–severe OSA	12	Nasal insufflation (precursor of HFNC), max 20 L/min	Reduced AHI, inspiratory flow limitation, arousals and respiratory rate; effect comparable to prior CPAP titration	Retrospective CPAP comparison, no washout period, fixed flow, small sample
Hawkins et al. [52]	Case series	Children with persistent OSA and CPAP intolerance	10	Overnight HFNC titration	OAHI 11.1 to 2.1 events/h; ODI and SpO_2_ improved	Small sample, selection bias, no CPAP comparator
Amaddeo et al. [50]	Prospective observational	Severe OSA with CPAP non-adherence	8	Home HFNC	5/8 achieved >4 h/night; mean ± SD AHI 2 ± 2 events/h in adherent patients	Small sample, short follow-up, no assessment of home adherence
Fishman et al. [26]	Randomized crossover trial	Obesity and/or medically complex children with moderate–severe OSA	18	HFNC vs. CPAP during PSG	Similar improvements in OAHI, ODI and sleep efficiency	Short-term titration study
Chan et al. [55]	Prospective laboratory and randomized home crossover study	Children/adolescents with moderate–severe OSA	29 PSG; 22 home	HFNC vs. CPAP (3 months each)	Similar residual OAHI; CPAP greater improvement in SaO_2_ nadir	Small sample; crossover design
Ehrlich et al. [56]	Multicenter retrospective cohort	Children receiving home HFNC for multiple indications	75	Long-term home HFNC	High caregiver satisfaction; hospitalization days decreased.	Not designed to assess PSG efficacy for OSA
Kwok et al. [53]	Retrospective HFNC titration study	Infants with OSA	10	Overnight HFNC titration during PSG	Median OAHI decreased from 9.1 to 0.9 events/h; oxygen saturation nadir improved from 88% to 94%	Small retrospective cohort; patients requiring supplemental oxygen were excluded from the final analysis; no comparator
Ignatiuk et al. [51]	Retrospective cohort and PSG titration study	Infants and young children with moderate-to-severe OSA, including poor surgical candidates and children with persistent OSA or CPAP intolerance	22	HFNC titration during PSG; 19 prescribed home HFNC	Mean OAHI decreased from 28.9 to 2.6 events/h; among children with ongoing OSA at 12 months, 7 continued HFNC and 5 discontinued because of intolerance	Retrospective design; heterogeneous population; attrition; limited objective long-term adherence data

#### 2.1.3. Adherence, Tolerance, and Patient Experience

A central rationale for HFNC in pediatric sleep-disordered breathing is improved interface tolerability. Unlike CPAP, HFNC delivers heated, humidified gas through a soft nasal cannula and avoids the need for a tightly fitted mask [23,30]. This may reduce mask-related discomfort, skin injury, claustrophobia, air leak, and behavioral resistance, particularly in children with craniofacial differences, neurodevelopmental disorders, sensory sensitivities, or prior CPAP intolerance [50,52].

However, better tolerance cannot be assumed. Amaddeo et al. reported clinically meaningful nightly use in a subset of children who were nonadherent to CPAP, whereas Chan et al. found better self-reported adherence with CPAP than with HFNC in otherwise healthy older children and adolescents [50,55]. These divergent findings suggest that acceptance of HFNC is highly phenotype- and age-dependent, and that a less intrusive interface does not necessarily translate into superior long-term adherence.

Reported adverse effects have generally been mild and include cannula displacement, nasal discomfort or dryness, skin irritation, epistaxis, restlessness, and caregiver burden related to equipment setup and circuit management [23,51]. Serious adverse events appear uncommon in published pediatric cohorts with OSA, although long-term safety data remain limited. Emergent central apneas have also been reported during HFNC titration in some children, possibly related to changes in ventilation or ventilatory control; their clinical significance remains uncertain but supports the need for polysomnographic titration and careful follow-up [26,57].

Overall, HFNC appears acceptable and feasible for selected children, but adherence remains heterogeneous and is not reliably captured by current devices. Future studies should prioritize objective adherence monitoring, caregiver-reported burden, patient preference, and standardized reporting of adverse events.

## 3. Discussion

HFNC has emerged as a promising but still incompletely defined therapy for pediatric sleep-disordered breathing. The available literature suggests that HFNC can reduce obstructive respiratory events and improve oxygenation in selected children, particularly those with persistent OSA, medical complexity, young age, or intolerance of conventional positive airway pressure. However, the evidence base remains limited with ongoing gaps in the existing evidence for the use of HFNC in children. Most studies are small, single-center investigations with heterogeneous populations, variable inclusion criteria, and differing titration protocols. Moreover, many studies evaluate short-term polysomnographic response, making it difficult to determine whether acute improvements translate into sustained benefit during long-term home use [25,26,50,51,55]. The titration of HFNC is a major challenge. Unlike CPAP, where pressure can be systematically adjusted and prescribed, HFNC is titrated based on flow in Liters per minute and the relationship between flow, generated pressure, leak, body size, airway anatomy, mouth position, and treatment response remains incompletely understood. As a result, the optimal HFNC “dose” for different pediatric phenotypes is unknown. Standardized titration protocols are needed, particularly because HFNC may be effective in some children at relatively modest flows, whereas others may require higher flows that may be less well tolerated or still insufficient to control obstruction.

Monitoring represents another important barrier to clinical implementation. Contemporary CPAP platforms provide objective adherence downloads, leak estimates, pressure information, and, in many devices, residual event indices. In contrast, currently available HFNC systems provide limited objective information regarding adherence or residual disease burden, forcing clinicians to rely on caregiver report which is variably accurate. Additionally, titration for HFNC is ideally done during level 1 polysomnography to evaluate the effectiveness of treatment. This presents an ongoing challenge internationally given the limited access to diagnostic pediatric sleep centers worldwide.

These limitations affect longitudinal follow-up, remote monitoring, treatment optimization, and the ability to distinguish true treatment failure from inadequate use.

Practical device-related issues also require consideration. Most HFNC systems currently used in pediatric sleep medicine were originally developed for respiratory support rather than long-term sleep applications. Compared with CPAP devices, they are generally larger, less portable, dependent on active humidification and regular water replacement, and require continuous electrical power without integrated battery-supported operation except for ventilators with integrated HFNC. Device costs, disposable circuits, nasal interfaces, humidification chambers, and other consumables may not be consistently covered by publicly funded programs or private insurance, creating inequities in access to treatment and long-term sustainability. These factors may limit travel outside the home dependent on the hours of usage in 24 h by the child, overnight respite care, the ability to remain at home during power outages, and the broader integration into family life.

Important uncertainties also remain regarding patient selection and clinically meaningful outcomes. Although HFNC can improve OSA severity and oxygenation in selected children, predictors of response are poorly defined. It remains unclear which phenotype of children including their anatomical, physiological, or clinical characteristics are most likely to benefit from HFNC, and which children require CPAP or Bi-level Positive Airway Pressure (BPAP) or other treatment strategies instead. Similarly, little is known regarding the effects of HFNC on neurocognitive outcomes, cardiovascular health, blood pressure, metabolic function, daytime functioning, or quality of life beyond the short-term.

Finally, the availability of long-term safety data remains limited. Serious adverse events appear uncommon in published pediatric cohorts, but the clinical significance of emergent central apneas, the effects of chronic high-flow therapy on ventilatory control of breathing, and the safety of prolonged home use require further study. Collectively, these limitations suggest that HFNC should currently be viewed as a second-line treatment for carefully selected children rather than a replacement for pressure-based therapy.

## 4. Conclusions

HFNC has emerged as a promising therapeutic option for selected children with sleep-disordered breathing, particularly those who are unable to tolerate conventional positive airway pressure therapy. Current evidence suggests that HFNC can improve obstructive respiratory events and oxygenation through a combination of flow-dependent positive airway pressure generation, dead-space washout, and enhanced ventilatory efficiency. However, important uncertainties remain regarding optimal patient selection, titration strategies, long-term effectiveness, objective monitoring, and comparative effectiveness relative to CPAP. As the evidence base continues to evolve, HFNC should be considered a complementary therapy within the pediatric sleep medicine armamentarium rather than a universal substitute for pressure-based treatment.

### Future Directions

Future research should move the field from feasibility towards implementation science and comparative effectiveness studies. Pragmatic multicenter studies are needed to evaluate whether HFNC improves outcomes that matter to children and families, including sustained treatment use, symptom burden, quality of life, daytime functioning, neurocognition, blood pressure, cardiometabolic health, and caregiver burden. Standardized reporting of adverse events, emergent central apneas, and treatment discontinuation will also be essential.

A priority is the development of pediatric HFNC titration and monitoring standards. Future studies should define age- and size-appropriate flow ranges, identify objective markers of adequate treatment response, and determine when HFNC failure should prompt transition to CPAP or BPAP. Incorporating physiological outcomes such as hypoxic burden, pulse rate response, sleep architecture, gas exchange, and ventilatory control may help determine whether improvements in OAHI reflect meaningful clinical benefit.

Technology must also evolve for HFNC to become a practical long-term sleep therapy. HFNC platforms designed for sleep medicine should ideally include objective usage data, leak or interface monitoring, flow-delivery verification, remote data access, battery-supported operation, and more portable designs. Although newer commercial platforms may address some usability barriers, their impact on adherence, home monitoring, clinical outcomes, cost, and equitable access in pediatric sleep-disordered breathing remains to be evaluated.

Finally, ongoing prospective multicenter studies may help clarify the role of HFNC within pediatric OSA treatment algorithms. Rather than evaluating HFNC simply as a more comfortable substitute for CPAP, future research should define its role as a distinct therapy with specific mechanisms, indications, limitations, and implementation requirements.

## Data Availability

No new data were created or analyzed in this study. Data sharing is not applicable to this article.
